# Comprehensive analysis of the overall codon usage patterns in equine infectious anemia virus

**DOI:** 10.1186/1743-422X-10-356

**Published:** 2013-12-20

**Authors:** Xin Yin, Yuezhi Lin, Weigang Cai, Ping Wei, Xiaojun Wang

**Affiliations:** 1 Division of Livestock Infectious Diseases, State Key Laboratory of Veterinary Biotechnology, Harbin Veterinary Research Institute, Chinese Academy of Agricultural Sciences, Harbin 150001, PR China; 2 College of Veterinary Medicine, Northeast Agricultural University, Harbin 150030, PR China

**Keywords:** Equine infectious anemia virus (EIAV), Codon usage bias, Evolution

## Abstract

**Background:**

Equine infectious anemia virus (EIAV) is an important animal model for understanding the relationship between viral persistence and the host immune response during lentiviral infections. Comparison and analysis of the codon usage model between EIAV and its hosts is important for the comprehension of viral evolution. In our study, the codon usage pattern of EIAV was analyzed from the available 29 full-length EIAV genomes through multivariate statistical methods.

**Finding:**

Effective number of codons (ENC) suggests that the codon usage among EIAV strains is slightly biased. The ENC-plot analysis demonstrates that mutation pressure plays a substantial role in the codon usage pattern of EIAV, whereas other factors such as geographic distribution and host translation selection also take part in the process of EIAV evolution. Comparative analysis of codon adaptation index (CAI) values among EIAV and its hosts suggests that EIAV utilize the translational resources of horse more efficiently than that of donkey.

**Conclusion:**

The codon usage bias in EIAV is slight and mutation pressure is the main factor that affects codon usage variation in EIAV. These results suggest that EIAV genomic biases are the result of the co-evolution of genome composition and the ability to evade the host’s immune response.

## Findings

### Introduction

Equine infectious anemia virus (EIAV) is an important nonprimate enveloped virus, of the retrovirus family, lentivirus genus, along with the human immunodeficiency virus (HIV), simian immunodeficiency virus (SIV) [1]. Among the lentiviruses, EIAV is the least complex lentivirus including only 6 genes. In addition to the *gag, pol and* e*nv* genes coding for the structural and enzymatic proteins coded by *gag, pol and env*, EIAV also contains three accessory genes: *tat*, r*ev* and *S2*[2]. The host range of EIAV is reported to include all members of the *Equidae*, while susceptible to infection, donkeys do not develop clinical EIA and lower amounts of plasma associated virus [3].

It is well known that the redundancy of the genetic code allows for multiple codons to encode for a single amino acid, resulting in codon usage biases in genes [4]. The non-random usage of synonymous codons is crucial for the efficient protein translation and correct folding. Indeed, mutation pressure and natural selection are thought to be two major forces that drive the codon usage bias away from an equal usage among genes in different organisms [5]. Understanding the extent and causes of biases in codon usage is important for the comprehension of the pathogen evolution and the relationship between pathogens and the immune response [6].

Recent efforts to understand codon usage biases in viruses have primarily focused on the hepatitis A virus [7,8], West Nile virus [9], foot-and-mouth disease virus [10], influenza virus [11], and HIV [12-14]. To date, although the remarkable adenine (A)-richness of the EIAV genome was already discovered several decades ago [15], few codon usage analyses have been performed on EIAV genome. To gain insight into the characteristics of the viral genome, the synonymous codon usage pattern and the correlation between the codon usage pattern of EIAV and its hosts were investigated in our study.

## Methods

### Sequence data

The complete genome sequences of 29 EIAV strains were obtained from the National Center for Biotechnology Information (NCBI) (http://www.ncbi.nlm.nih.gov/Genbank/). The detailed information about the viruses is listed in Additional file 1: Table S1.

### Codon usage analysis

Each nucleotide content and each nucleotide content at the third site of the codon in the EIAV coding sequence were calculated using MEGA4 software. The dinucleotides of the EIAV genome were analyzed by DAMBE software. The relative synonymous codon usage (RSCU) values for EIAV were calculated as previously described [16]. The effective number of codons (ENC), was used to quantify deviations from the expected random codon usage of EIAV ORFs [17]. The ENC values range from 20 to 61, and a low ENC value indicates a strong codon usage bias.

The codon adaptation index (CAI) was used to estimate the adaptation of EIAV to host codons. When the CAI value is much closer to 1, the gene expression level is much higher. The CAI was calculated to compare a given codon usage to a predefined reference set, using the CAIcal approach (available at: http://genomes.urv.es/CAIcal). The synonymous codon usage data for the viral hosts were obtained from the codon usage database (http://www.kazusa.or.jp/codon/)[18].

### Principal component analysis

Principal component analyses (PCA) were performed to analyze the major trend in the codon usage model among the different EIAV strains. Each ORF is represented as a 59-dimensional vector and each dimension corresponds to the RSCU value of one sense codon, excluding the codons of AUG, UGG and terminal codons. The major trend within a dataset can be determined using measure of relative inertia and genes ordered according to their position along the axis of major inertia [19].

## Results and discussion

### Synonymous codon usage in EIAV

The overall base composition of different EIAV strains was nonrandom, and the U% and A% were higher than the C% and G% (Table 1). The EIAV genome which was rich in A may possess viral tactics for escaping from the antiviral activity of apolipoprotein B mRNA-editing enzyme-catalytic polypeptide 3 (APOBEC3) [20,21]. To investigate whether these 29 EIAV strains display similar codon usage biases, the ENC values were calculated. The obtained ENC values varied from 38.10 to 49.71 with a mean of 43.61 ± 3.28. One possible explanation for the weak synonymous codon bias of EIAV was that the weak codon bias is essential to increasing the translational accuracy and efficiency. To further analyze the extent of codon usage bias in EIAV, the overall RSCU values for the 59 sense codons were calculated. Almost all extremely highly preferred codons among the strains were A-ended or U-ended codons (Table 2). In addition, AA was the most common dinucleotide in EIAV, while CG dinucleotides were significant suppressed in the genome of EIAV. The result revealed that CpG suppression may be a factor that shapes the synonymous codon usage of EIAV genome.

**Table 1:** The overall nucleotide contents and nucleotide contents at the synonymous third position of sense codons in EIAV genome

**No. of genomes analyzed**	**T (%) ± std**	**C (%) ± std**	**A (%) ± std**	**G (%) ± std**	**T 3(%) ± std**	**C3 (%) ± std**	**A3 (%) ± std**	**G3 (%) ± std**
**29**	**25.39 ± 0.32**	**15.57 ± 0.33**	**37.09 ± 0.32**	**21.96 ± 0.24**	**29.62 ± 0.78**	**11.52 ± 0.58**	**38.01 ± 0.83**	**20.85 ± 0.63**

**Table 2:** Codon usage in EIAV genomes and its hosts

**AA**	**Codon**	**RSCU**			**AA**	**Codon**	**RSCU**		
		**EIAV**	**Horse**	**Donkey**			**EIAV**	**Horse**	**Donkey**
**Phe**	UUU	1.48	0.82	0.89	**Cys**	UGU	1.50	0.90	0.62
	UUC	0.52	1.18	1.11		UGC	0.50	1.10	1.38
**Asn**	AAU	1.50	0.84	0.66	**Arg**	CGU	0.17	0.36	0.48
	AAC	0.50	1.16	1.34		CGC	0.18	1.14	0.78
**Lys**	AAA	1.54	0.80	0.78		CGA	0.20	0.60	0.72
	AAG	0.46	1.20	1.22		CGG	0.31	1.08	0.60
**Leu**	UUA	2.19 2.06	0.30	0.24		AGA	3.09	1.30	1.50
	UUG	1.29	0.70	0.84		AGG	2.05	1.34	1.86
	CUU	0.58	0.74	0.78	**Ser**	UCU	1.42	1.08	1.08
	CUC	0.46	1.32	1.62		UCC	0.73	1.44	1.44
	CUA	0.85	0.36	0.18		UCA	1.41	0.78	0.84
	CUG	0.62	2.58	2.34		UCG	0.12	0.48	0.24
**Pro**	CCU	2.04	1.20	0.84		AGU	1.71	0.84	1.08
	CCC	0.40	1.28	1.60		AGC	0.61	1.50	1.26
	CCA	1.44	0.96	1.04	**Asp**	GAU	1.35	0.84	0.87
	CCG	0.12	0.44	0.52		GAC	0.65	1.16	1.13
**Thr**	ACU	1.62	0.92	0.84	**Glu**	GAA	1.25	0.76	0.84
	ACC	0.42	1.60	1.76		GAG	0.75	1.24	1.16
	ACA	1.79	0.96	0.80	**His**	CAU	1.45	0.80	0.88
	ACG	0.17	0.52	0.60		CAC	0.55	1.20	1.12
**Val**	GUU	0.74	0.60	0.64	**Gln**	CAA	1.28	0.52	0.84
	GUC	0.37	1.08	1.40		CAG	0.72	1.48	1.16
	GUA	1.96	0.36	0.28	**Gly**	GGU	0.64	0.64	0.88
	GUG	0.93	1.96	1.64		GGC	0.38	1.44	1.44
**Ala**	GCU	1.72	1.08	1.20		GGA	2.05	0.94	0.82
	GCC	0.44	1.72	1.76		GGG	0.92	0.98	0.86
	GCA	1.60	0.76	0.76	**Ile**	AUU	1.04	0.93	0.57
	GCG	0.24	0.44	0.28		AUC	0.45	1.65	1.95
**Tyr**	UAU	1.45	0.76	0.64		AUA	1.51	0.52	0.48
	UAC	0.55	1.24	1.36					

### The effect of mutation pressure on the codon usage of EIAV

**Figure 1:**
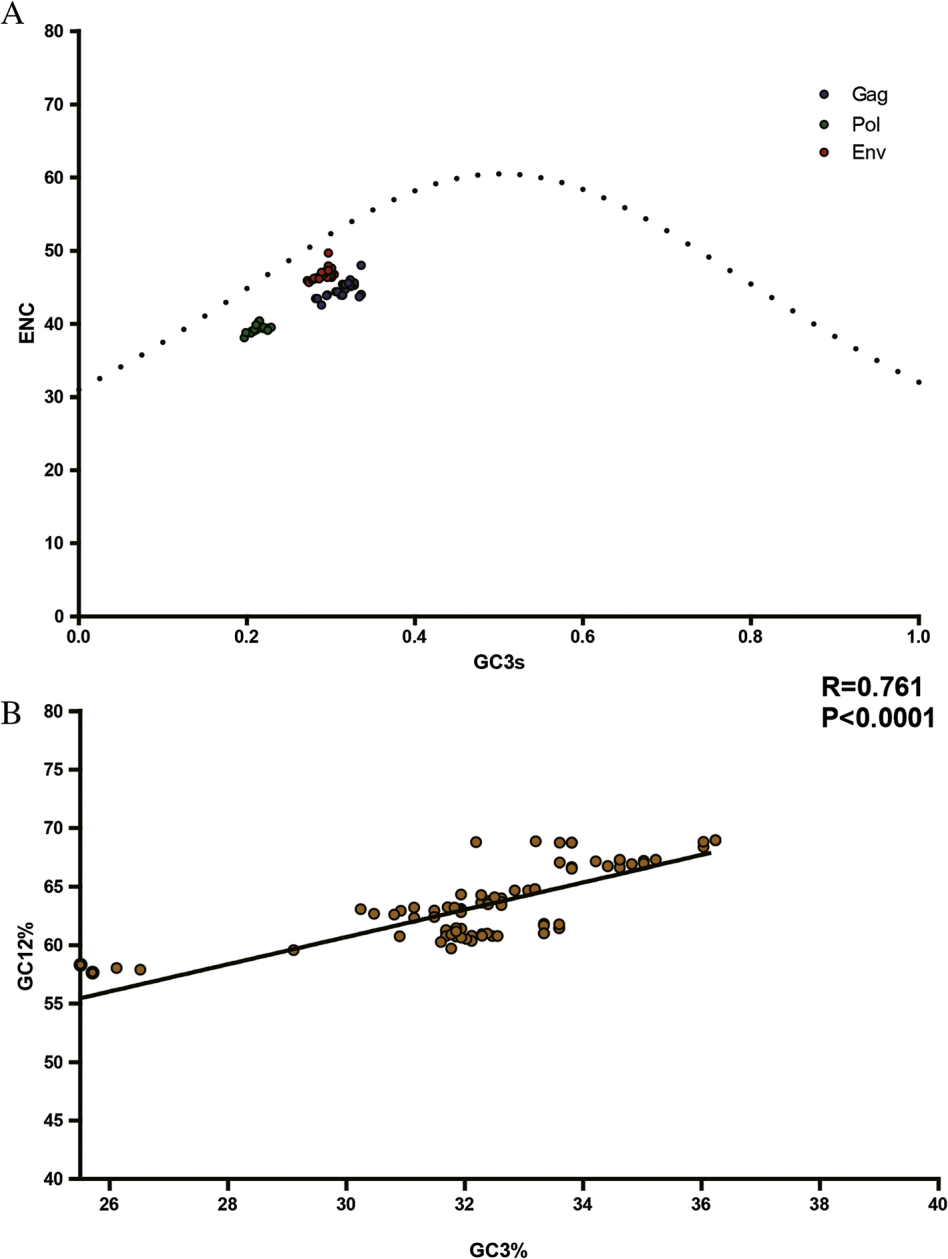
**The main forces that affect the codon usage of EIAV. (A)** The relationship between the effective number of codons (ENC) and the GC content of the third codon position (GC_3_). The continuous curve represents the expected curve between ENC value and GC_3_% in the absence of selection. All of spots lie below the expected curve. (ENC, GC_3_%) values of *gag*, *pol* and *env* were indicated by *blue plot*, *green plot*, and *red plot* respectively. **(B)** Correlation between G + C content at the first and second codon positions (GC_12_%) and that at synonymous third codon positions (GC_3_%). The line represents the correlation curve generated by the correlation analysis.

The ENC-plot analysis (ENC value plotted against the GC_3s_ content) was performed to further investigate the patterns of synonymous codon usage. We found that all of the spots lie slightly below the expected curve, indicating that mutational pressure was the main factor for shaping the codon usage bias of EIAV (Figure 1A). To further identify the role of mutation pressure from the virus itself or by natural selection pressure in shaping the codon usage pattern of EIAV, a correlation analysis was used to analyze the relationships among the G + C content at the first and second codon positions (GC_12_%) and that at the synonymous third codon positions (GC_3_%). A highly significant correlation was observed (r =0.761, P < 0.001), indicating that the mutation pressure dominated over the natural selection pressure in shaping the coding sequence’s composition (Figure 1B).

### Genetic relationship based on synonymous codon usage in EIAV

**Figure 2:**
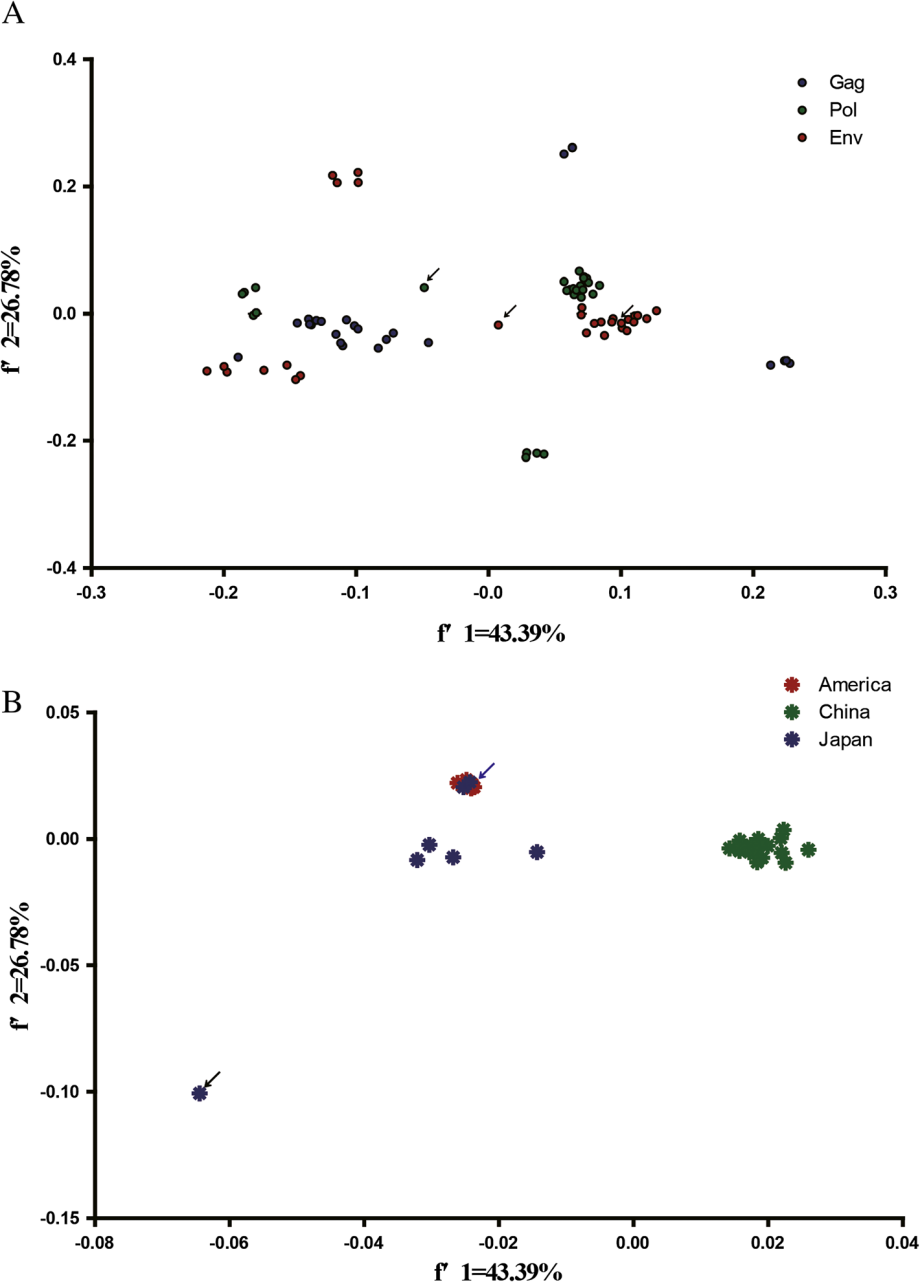
**Genetic relationship based on synonymous codon usage in EIAV. (A)** A plot of the values of the first axis and the second axis of *gag*, *pol*, and *env* in principle component analysis. EIAV field strain Miyazaki2011-A was indicated by *black arrow*. **(B)** A plot of the values of the first axis and the second axis of EIAV strains isolated from China (*green*), Japan (*blue*), and America (*red*) in principle component analysis. EIAV field strain Miyazaki2011-A was indicated by *black arrow*, EIAV strains V70 and V26 was indicated by *blue arrow*.

The first principal component (f'1) which can account for 43.39% of the total variation has a substantial impact on the total variation in the codon usage pattern. In addition, a plot of the *f*'1 and *f*'2 of the *gag, pol, and env* ORFs in EIAV was drawn (Figure 2A). The plots for the different structural proteins were generally separated from each other. This phenomenon implied that the functions of the viral protein were likely related to the codon usage pattern.

It has been reported that a strong pattern of geographic clustering is observed for EIAV, with a significant correlation between phylogroups of isolates and major geographic regions [22]. Based on the potential for the geographical factors in influencing EIAV evolution, a plot of *f*'1 and *f*'2 was performed according to the geographic distribution. The plots for EIAV isolated from China, Japan, and America were generally divided into three groups, implying that the EIAV isolated from the three countries evolved independently after diverging from a common ancestor (Figure 2B). In addition, we cannot ignore that the plots for EIAV strains V70 and V26 were clustered together with the strains isolated from America. The origin of these strains still remains controversial [23,24]. Our data demonstrated that these EIAV strains have an American ancestry. Notably, the EIAV Miyazaki2011-A plot was far from the plots of the other strains. Recent reports showed that this EIAV strain was unlikely derived as a result of genomic recombination events and constituted a separate monophyletic group [24]. It is interesting to identify the potential origin of this novel EIAV isolate.

### Comparative analysis of the codon usage between EIAV and host cells

The synonymous codon usage pattern of EIAV tended to differ from that of horse and donkey (Table 2 and Additional file 2: Figure S1). To further investigate whether the frequency of codon usage between EIAV and its hosts might have a close relationship with the viral proteins’ expression levels, the CAI were calculated using the horse and donkey codon usage as reference sets [25]. A mean CAI of 0.655 ± 0.020 was obtained for the EIAV ORFs in relation to horse codon usage reference set. A mean CAI of 0.593 ± 0.021 was obtained for the EIAV ORFs in relation to the donkey codon usage reference set. There was a trend for a lower CAI for EIAV in relation to donkey, with the consequent lower efficiency of protein synthesis in donkey. This phenomenon reflected that the interplay of codon usage between EIAV and its hosts may influence viral fitness, survival and evolution.

In conclusion, our comprehensive analysis of the codon usage patterns in EIAV has provided a basic understanding about some of the evolutionary information of EIAV. However, there were some limitations to this study. The sample size was relatively small and may not be fully representative of EIAV. More studies should be carried out to confirm the conjecture.

## Competing interests

The authors declare that they have no competing interests.

## Authors’ contributions

XY performed the experiments and wrote the first draft of the paper, in collaboration with XJW and PW. YZL and WGC analyzed the data and drafted the manuscript. All authors read and approved the final manuscript.

## Supplementary Material

Additional file 1: Table S1 List of the information about the 29 EIAV genomes used in this study. Click here for file

Additional file 2: Figure S1 The ENC-plot for mean codon usage in horse and donkey. The continuous curve represents the expected curve between ENC value and GC_3_% in the absence of selection. (ENC, GC_3_%) values of *horse and donkey* were indicated by *red plot* and *blue plot* respectively. Click here for file
