# Supplementary material for: Comprehensive analysis of the overall codon usage patterns in equine infectious anemia virus
Source: Virol J. 2013 Dec 20;10:356. doi: 10.1186/1743-422X-10-356 (PMC3878193; doi:10.1186/1743-422X-10-356)
Supplement: Additional file 1: Table S1 — List of the information about the 29 EIAV genomes used in this study. [file 1743-422X-10-356-S1.doc]

Table S1 List of the information about the 29 EIAV genomes used in this study

| **No.** | **Strain** | **Country of isolation** | **Accession no.** |
| --- | --- | --- | --- |
| 1 | Equine infectious anemia virus strain Vaccine strain | China | AF327878 |
| 2 | Equine infectious anemia virus strain Liaoning strain | China | AF327877 |
| 3 | Equine infectious anemia virus isolate H3 | Japan | JX480634 |
| 4 | Equine infectious anemia virus isolate F4 | Japan | JX480633 |
| 5 | Equine infectious anemia virus isolate F3 | Japan | JX480632 |
| 6 | Equine infectious anemia virus isolate F2 | Japan | JX480631 |
| 7 | Equine infectious anemia virus strain Miyazaki2011-A | Japan | JX003263 |
| 8 | Equine infectious anemia virus isolate DLV18-8 | China | HM141923 |
| 9 | Equine infectious anemia virus isolate DLV9-7 | China | HM141922 |
| 10 | Equine infectious anemia virus isolate DLV5-10 | China | HM141921 |
| 11 | Equine infectious anemia virus isolate DLV2-6 | China | HM141920 |
| 12 | Equine infectious anemia virus isolate DV31-2 | China | HM141919 |
| 13 | Equine infectious anemia virus isolate DLV15-16 | China | HM141918 |
| 14 | Equine infectious anemia virus isolate DLV19-9 | China | HM141917 |
| 15 | Equine infectious anemia virus isolate DLV10-19 | China | HM141916 |
| 16 | Equine infectious anemia virus isolate DLV8-2 | China | HM141915 |
| 17 | Equine infectious anemia virus isolate DLV7-18 | China | HM141914 |
| 18 | Equine infectious anemia virus isolate DLV3-A | China | HM141913 |
| 19 | Equine infectious anemia virus isolate DV117 | China | HM141912 |
| 20 | Equine infectious anemia virus isolate DLV35-20 | China | HM141911 |
| 21 | Equine infectious anemia virus isolate DV10-3 | China | HM141910 |
| 22 | Equine infectious anemia virus isolate DV3-5 | China | HM141909 |
| 23 | Equine infectious anemia virus clone p19wenv17 | America | AF028232 |
| 24 | Equine infectious anemia virus clone p19wenv16 | America | AF028231 |
| 25 | Equine infectious anemia virus clone EIAVuk | America | AF016316 |
| 26 | Equine infectious anemia virus isolate WSU5 | America | AF247394 |
| 27 | Equine infectious anemia virus strain Wyoming | America | AF033820 |
| 28 | Equine infectious anemia virus isolate V26 | Japan | AB008197 |
| 29 | Equine infectious anemia virus isolate V70 | Japan | AB008196 |
