# Supplementary figures and images for: Comprehensive analysis of the overall codon usage patterns in equine infectious anemia virus
Source: Virol J. 2013 Dec 20;10:356. doi: 10.1186/1743-422X-10-356 (PMC3878193; doi:10.1186/1743-422X-10-356)

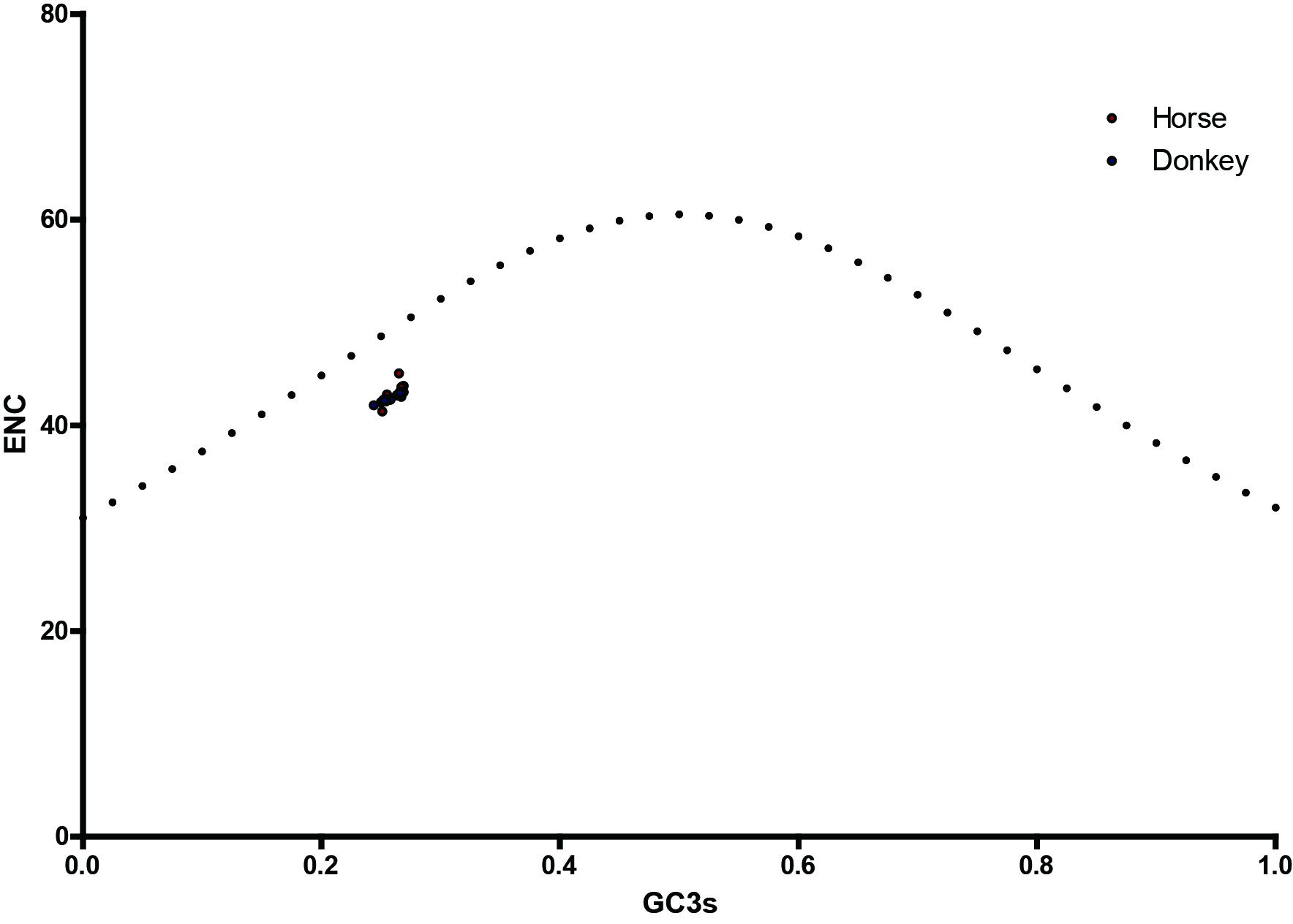

Supplement: Additional file 2: Figure S1 — The ENC-plot for mean codon usage in horse and donkey. The continuous curve represents the expected curve between ENC value and GC3% in the absence of selection. (ENC, GC3%) values of horse and donkey were indicated by red plot and blue plot respectively. [file 1743-422X-10-356-S2.jpeg]
